# Is COVID-19 incriminated in new onset type 2 diabetes mellitus in Lebanese adults?

**DOI:** 10.1186/s13104-023-06454-4

**Published:** 2023-08-18

**Authors:** Rose Mary Jabbour, Souheil Hallit, Rita Saliby, Abed El Karim Baydoun, Nancy Nakhoul

**Affiliations:** 1 School of Medicine and Medical Sciences, Holy Spirit University of Kaslik, P.O. Box 446, Jounieh, Lebanon; 2 Applied Science Research Center, Applied Science Private University, Amman, Jordan; 3 Research Department, Psychiatric Hospital of the Cross, Jal Eddib, Lebanon; 4 Faculty of Medicine, University of Balamand, Koura, Lebanon

**Keywords:** COVID-19 infection, Coronavirus, Diabetes mellitus, New onset diabetes

## Abstract

**Background:**

The effects of COVID-19 on the organism are still being investigated, especially after the transformation of this virus from a respiratory disease in its first appearance to a multi-organ disease that can affect nearly all systems and organs including the endocrinological system. The objective of the study was to find an association between COVID-19 infection and new onset type 2 diabetes in Lebanese adults.

**Methods:**

A retrospective case–control study (2019–2022) included 200 subjects, 100 cases with new onset diabetes and 100 controls recruited from endocrinology clinics in rural and suburban located regions of Lebanon. Univariate and multivariate logistic regression were performed.

**Results:**

Older age (aOR = 1.07; 95% CI 1.03–1.12), higher BMI (aOR = 1.32; 95% CI 1.17–1.48), having been infected with COVID-19 (aOR = 2.38; 95% CI 1.001–5.68) and having a family history of diabetes (aOR = 11.80; 95% CI 4.23–32.87) were significantly associated with higher odds of having new onset type 2 diabetes after adjusting for multiple risk factors.

**Conclusion:**

In addition to the traditional risk factors for developing type 2 diabetes, a recent COVID-19 infection was associated with the new onset DM in our study. Subsequently screening for diabetes should be strongly recommended for patients post COVID-19 infection.

**Supplementary Information:**

The online version contains supplementary material available at 10.1186/s13104-023-06454-4.

## Introduction

Diabetes mellitus type 2 (DMT2) is among the most challenging public health concerns nowadays, affecting more than 537 million persons [1], which might have a negative impact on the patient’s quality of life [2]. Its prevalence is 10.5% worldwide [1], 7.95% in Lebanon [3], with 9.5% undiagnosed cases [4]. The main cause behind this rise in the prevalence of DMT2 is the sedentary lifestyle and inappropriate eating attitudes [5], as well as poor knowledge about the disease [6].

In December 2019, a new form of pneumonia emerged in Wuhan city, China. The highly contagious virus causing this type of pneumonia was termed severe acute respiratory syndrome coronavirus 2 causing the SARS-COVID 19 disease [7]. This virus led to a change in the world’s demographics since it took away the lives of millions of people [8]. COVID-19 infection led to a wide range of new, returning, or ongoing health problems that people experience after being infected [9]. The outcomes of the infection range from mild forms of infection with spontaneous resolution within few days or weeks [10], to severe forms of infection that require further measures of treatment such as Intensive Care Unit admission, oxygen supplementation, corticosteroids, and intubation, with tendency of having multi-organ failure [11].

The duration of COVID-19 symptoms is somehow independent of the infection itself, where many patients experience long-term effects of the virus on their organs; this condition is termed “Post-COVID-19 conditions” or “Long COVID-19” [10]. Four weeks after recovery from the infection, post COVID-19 conditions can be identified [10]. The most common post infection complications are respiratory, cardiac, neurological, digestive and metabolic [9]. The cause of these complications is still unknown, with various hypotheses being formulated regarding the mechanism of action of these different conditions [12].

It is known that Diabetes Mellitus Type 2 is associated with different viral infections (e.g. Influenza, Dengue…), which is also an evidence that COVID-19 could also be associated with new onset of diabetes [13]. The mechanisms suggested for the development of Diabetes Mellitus post COVID-19 infection are (1) either by the affinity of the virus to bind to target cells and therefore modifying the normal function of pancreatic cells (i.e. binding to Angiotensin Converting Enzyme (ACE)-2 receptors and consequently causing accumulation angiotensin 2, which causes glucose dysregulation) [14] or (2) by direct damage to beta islet cells and therefore interfering with insulin secretion [15]. The effect on glucose regulation can also be extra-pancreatic through adipose tissues, another target of the COVID-19 virus, which causes insulin resistance and chronic inflammation [16].

A rise in subjects developing both types of diabetes 1 and 2 after COVID-19 infection was observed in comparison to the number of new cases of diabetes reported before December 2019 [17]. Signs of new onset diabetes appeared in the first few months of the pandemic and are still ongoing [17]. Compared to uninfected people, a large study published in March 2022 in the United States estimated that any positive COVID-19 test increases a person’s probability of developing any type of diabetes by 40% in the upcoming year [17].

In Lebanon, as of March 2, 2023, there were 1,232,063 confirmed cases of COVID-19, with 10,832 deaths (0.87% of total confirmed cases) [18]. Previous studies showed a positive link between COVID-19 infection and new-onset diabetes [19, 20]. Since no previous studies tackling this topic were conducted in Lebanon, and little is known from an endocrinological point of view [12], the aim of this study was to identify a possible association between COVID-19 infection and new onset diabetes in a sample of Lebanese adults.

## Methods

### Study design and participants

This retrospective case–control study included patients who were seen several endocrinology clinics between February 2020 and February 2023, covering a wide area of rural and sub-urban regions of Lebanon (mainly Mount Lebanon and North Lebanon governorates). Information from medical records of the patients were collected. Controls were recruited from the same clinics; they were matched with cases for age and sex. Participants included in this study met the following criteria: Lebanese and aged above 18. Excluded were those under 18 years of age, patients with prediabetes, a confirmed or known diagnosis of diabetes and those with a new onset diabetes before February 2020.

### Minimum sample size

Since no previous studies tackled this objective, we conducted a pilot study that enrolled 100 cases of new-onset diabetes and 100 non-diabetic controls.

### Data collection

The data collection sheet can be found as Additional file 1. Demographic data, date of COVID-19 infection, need of hospital stay during COVID-19 infection, comorbidities, family history of diabetes, date of diabetes diagnosis, and vaccination status were collected from medical records. The time between the date of infection and the date of diabetes diagnosis was reported. Treatment with corticosteroids during active COVID-19 infection was also assessed since steroids use is known for steroid- induced hyperglycemia [21].

### Statistical analysis

Analysis was carried out using the SPSS software v.25. The Chi-square was used to compare two categorical variables, whereas the Student t test was used to compare two means. A logistic regression was conducted afterwards, taking the presence vs. absence of new-onset diabetes as the dependent variable. Factors that showed a *p* < 0.25 in the bivariate analysis were considered as independent variables in the final model. *P* < 0.05 was deemed statistically significant.

## Results

Two hundred patients enrolled in the study, 100 new onset diabetes cases and 100 controls matched for age and gender. Their mean age was 52.21 ± 12.80 years, with 25.0% females. The mean duration between the date of COVID-19 infection and the date of diabetes diagnosis was 6.2 ± 3.3 months.

### Bivariate analysis

The results of the bivariate analysis are summarized in Table 1. A significantly higher percentage of patients with new onset type 2 diabetes had COVID-19 infection, was not admitted to the ICU, was nonsmokers, did have hypertension, did have a family history of diabetes, did receive corticosteroids during the COVID-19 infection, and had not received the COVID-19 vaccine. Furthermore, a higher mean BMI was found in patients with new onset type 2 diabetes.

**Table 1:** Bivariate analysis of factors associated with the new onset of type 2 diabetes

	No	Yes	*p*
Gender			0.511
Male	86 (76.8%)	64 (72.7%)	
Female	26 (23.2%)	24 (27.3%)	
COVID-19 infection			**0.001**
No	60 (53.6%)	26 (29.5%)	
Yes	52 (46.4%)	62 (70.5%)	
Admission to the ICU			** < 0.001**
No	111 (99.1%)	74 (84.1%)	
Yes	1 (0.9%)	14 (15.9%)	
Coronary artery disease			0.463
No	91 (81.3%)	67 (77.0%)	
Yes	21 (18.8%)	20 (23.0%)	
Dyslipidemia			0.059
No	59 (52.7%)	58 (65.9%)	
Yes	53 (47.3%)	30 (34.1%)	
Smoking			**0.020**
No	49 (43.8%)	52 (60.5%)	
Yes	63 (56.3%)	34 (39.5%)	
Hypertension			**0.044**
No	79 (70.5%)	50 (56.8%)	
Yes	33 (29.5%)	38 (43.2%)	
Family history of diabetes			** < 0.001**
No	101 (90.2%)	55 (63.2%)	
Yes	11 (9.8%)	32 (36.8%)	
Use of corticosteroids			** < 0.001**
No	110 (98.2%)	68 (77.3%)	
Yes	2 (1.8%)	20 (22.7%)	
Vaccination			**0.021**
No	12 (10.7%)	20 (22.7%)	
Yes	100 (89.3%)	68 (77.3%)	
Age (in years)	50.85 ± 13.53	53.94 ± 11.65	0.090
Body Mass Index (kg/m^2^)	26.89 ± 3.65	30.20 ± 4.69	** < 0.001**

Significant *p* values are shown in bold

### Multivariable analysis

Older age (aOR = 1.07; 95% CI 1.03–1.12), higher BMI (aOR = 1.32; 95% CI 1.17–1.48), having been infected with COVID-19 (aOR = 2.38; 95% CI 1.001–5.68) and having a family history of diabetes (aOR = 11.80; 95% CI 4.23–32.87) were significantly associated with higher odds of having new onset type 2 diabetes. In addition, a significant correlation was established between dyslipidemia (aOR = 0.41; 95% CI 0.18–0.95) and lower odds of developing new onset type 2 diabetes (Table 2).

**Table 2:** Logistic regression taking the presence vs absence* of new onset diabetes type 2 as the dependent variable (R^2^ = 0.542)

	*p*	aOR	95% CI
Age	**0.001**	1.07	1.03; 1.12
Body Mass Index	** < 0.001**	1.32	1.17; 1.48
COVID-19 infection (yes vs no*)	**0.050**	2.38	1.001; 5.68
Admission to ICU (yes vs no*)	0.183	5.03	0.47; 54.25
Dyslipidemia (yes vs no*)	**0.037**	0.41	0.18; 0.95
Smoking (yes vs no*)	0.267	0.64	0.29; 1.40
Hypertension (yes vs no*)	0.276	1.62	0.68; 3.84
Family history of diabetes (yes vs no*)	** < 0.001**	11.80	4.23; 32.87
Use of glucocorticoids during COVID-19 infection (yes vs no*)	0.060	5.45	0.93; 31.90
COVID-19 vaccine intake (yes vs no*)	0.369	0.59	0.19; 1.87

Numbers in bold indicate significant p values

## Discussion

Our study confirmed that COVID-19 infection is a strong independent factor associated with the development of new onset diabetes mellitus (NODM) after adjusting for other confounding variables; this result is in agreement with previous findings [18] that also showed a high association between COVID-19 and NODM; 70% of our patients who developed NODM got infected by COVID-19 prior to their diagnosis. This might be due to the effect of COVID-19 virus on glycemia regulation by causing immune response through cytokine storm and damaging islet cells, or by Angiotensin Converting Enzyme 2 (ACE-2) downregulation and accumulation of angiotensin 2, which impairs insulin secretion [18].

The mean duration between the date of COVID-19 infection and that of diabetes diagnosis was 6.2 ± 3.3 months; this time interval between COVID-19 infection and diabetes differed from the one shown in previous studies (NODM within 3 months [22] and 1 month after COVID-19 infection [23] respectively).

According to the results obtained from our study, higher Body Mass Index (BMI) was associated with higher odds of developing NODM post COVID-19, corroborating previous results [13]. This emphasizes obesity as a traditional risk factor for diabetes mellitus development in individuals without prior COVID-19 infection and additionally increasing the risk of NODM post COVID-19 infection. Adipose tissue acts as a source of entry for SARS-CoV-2 since it contains a significant amount of ACE-2 receptors, therefore adipose tissues can be widely affected by the virus [14, 18]. Adipocytes are very rich in cytokines; by causing stress on these adipocytes, COVID-19 infections aggravate chronic inflammation and hyperglycemia [14, 18]. Hyperglycemic patients with COVID-19 infection suffer from high levels of inflammatory markers compared to infected patients with normal glycemia [14, 18]. This was also noted in a study of Keerthi et al. which showed that higher BMI is a predictor of NODM [13].

In our study, the multivariable analysis confirmed that a family history of diabetes, a traditional risk factor of the disease, was associated with higher odds of new onset diabetes. Similar results were observed in a study done by Keerthi et al. which demonstrated that 14% of patients with a history of COVID-19 infection and new onset diabetes mellitus had positive family history [13].

Age was also significantly associated with higher odds of NODM in our study. In our study, the mean age of patients with new onset diabetes and COVID-19 (70%) was 49.9 years compared to 57.8 years in patients with new onset of diabetes without COVID-19 (30% of patients with NODM without COVID-19 infection) (data not shown). This finding highlights the possible role of COVID-19 in the development of NODM in younger patients, making COVID-19 infection as a possible predictor of young onset Diabetes [13].

Despite dyslipidemia being an element of metabolic syndrome a predisposing to diabetes [13], our results in the multivariable analysis showed that dyslipidemia decreased the odds of having new onset diabetes. This might be due to a selection bias where the controls were recruited from the same endocrinology clinic therefore, other than diabetes, their complaint is most likely related to thyroid problems or dyslipidemia.

### Limitations and strengths

Although our data collection involved many clinics from a wide region of Lebanon, other regions were not represented in our study, predisposing us to a selection bias. Some patients may have had preexisting diabetes that was not discovered or investigated before the infection. Despite including many known risk factors associated with DM, residual confounding bias might be present since not all factors associated with diabetes were considered in this study. Recall/information bias is possible since patients might have forgotten the exact date of infection.

The strengths of our study rely in the fact that it is the first in Lebanon and Middle East to tackle the association between COVID-19 infection and NODM, the large number of patients included, and the inclusion of multiple risk factors know to be associated with DM, which adds to the robustness of the results.

## Conclusion

Our study demonstrated that COVID-19 infection is a significant factor associated with the development of new onset diabetes. Subsequently screening for diabetes should be strongly recommended for patients post infection, specifically in those having common risk factors for diabetes such as age, genetic predisposition, obesity, high blood pressure, and other endocrine diseases [24]. This would allow early treatment to avoid potential hyperglycemia complications. The emergence of diabetes in COVID-19 pandemic imposes the question on whether this is a new type of atypical diabetes. Understanding the impact of COVID-19 on different systems can orient health professionals towards the possible outcomes of this infection on long-term basis. Evaluating risk factors, severity of the diseases, general data of each patients can further help prevent or limit comorbidities associated with this disease by screening patients, educating as well as preventing with different means (lifestyle, checkups, medications, etc.) the unfavorable outcomes of the COVID-19 infection**.**

### Supplementary Information

**Additional file 1: **Data sheet of the variables used in the data collection

## Data Availability

The authors do not have the right to share any data information as per their institutions policies. Data can be shared upon a reasonable request to the corresponding author.
