# Supplementary material for: Is COVID-19 incriminated in new onset type 2 diabetes mellitus in Lebanese adults?
Source: BMC Res Notes. 2023 Aug 18;16:176. doi: 10.1186/s13104-023-06454-4 (PMC10439538; doi:10.1186/s13104-023-06454-4)
Supplement: Supplementary file 1 — Additional file 1: Data sheet of the variables used in the data collection [file 13104_2023_6454_MOESM1_ESM.docx]

**Appendix 1: Data sheet of the variables used in the data collection.**

Gender: Male Female

Age (in years)

Height (in meters)

Weight (in Kg)

Smoking: No Yes

COVID-19 infection: No Yes

If yes, date of the COVID-19 infection:

COVID-19 vaccine: No Yes

Family History: No Yes

ICU admission: No Yes

Steroids use: No Yes

Hypertension: No Yes

Coronary artery disease: No Yes

Dyslipidemia: No Yes
